# New techniques: a roadmap for the development of HCC immunotherapy

**DOI:** 10.3389/fimmu.2023.1121162

**Published:** 2023-06-22

**Authors:** Dizhi Jiang, Xinyue Ma, Xun Zhang, Bo Cheng, Ruiqing Wang, Yuan Liu, Xinyu Zhang

**Affiliations:** Department of Radiation Oncology, Qilu Hospital of Shandong University, Cheeloo College of Medicine, Shandong University, Jinan, China

**Keywords:** hepatocellular carcinoma, immunotherapy, CRISPR, scRNA-seq, immune checkpoint inhibitor

## Abstract

Hepatocellular carcinoma (HCC) is one of the most common cancers worldwide. The absence of effective early diagnostic methods and the limitations of conventional therapies have led to a growing interest in immunotherapy as a novel treatment approach for HCC. The liver serves as an immune organ and a recipient of antigens from the digestive tract, creating a distinctive immune microenvironment. Key immune cells, including Kupffer cells and cytotoxic T lymphocytes, play a crucial role in HCC development, thus offering ample research opportunities for HCC immunotherapy. The emergence of advanced technologies such as clustered regularly interspaced short palindromic repeats (CRISPR) and single-cell ribonucleic acid sequencing has introduced new biomarkers and therapeutic targets, facilitating early diagnosis and treatment of HCC. These advancements have not only propelled the progress of HCC immunotherapy based on existing studies but have also generated new ideas for clinical research on HCC therapy. Furthermore, this review analysed and summarised the combination of current therapies for HCC and the improvement of CRISPR technology for chimeric antigen receptor T cell therapy, instilling renewed hope for HCC treatment. This review comprehensively explores the advancements in immunotherapy for HCC, focusing on the use of new techniques.

## 1 Introduction

The mortality rate attributed to liver cancer is projected to exceed one million by 2030 (1). The Global Cancer Observatory data reveals that in 2018, China accounted for 46.6% of newly diagnosed cases and 47.1% of deaths related to liver cancer (2). Hepatocellular carcinoma (HCC) and cholangiocarcinoma are the predominant forms of primary liver cancers, accounting for approximately 75% and 6% of cases, respectively (3).

Despite the availability of surgical resection or liver transplantation as treatment options for liver cancer, their effectiveness is limited due to high recurrence rates after resection and low surgical and transplant eligibility ratios because most patients are diagnosed at an advanced stage where curative treatment is not recommended (4, 5). Therefore, a combination of radiotherapy, chemotherapy, hormonal therapy, and targeted therapy is often necessary. Advancements have been made in chemotherapy and targeted therapy for HCC. For instance, the combination of gemcitabine and cisplatin as a first-line treatment is more effective than gemcitabine alone (6). Lenvatinib, approved in 2017, has shown an overall survival (OS) of 13.6 months and progression-free survival (PFS) of 7.4 months (7). Nevertheless, the overall outcomes remain discouraging. Immunotherapy has gained attention in the medical community considering its recent success in treating solid tumours, which have traditionally been considered immune-cold (8).

Since the initial testing of the first inhibitor of the immune checkpoint, cytotoxic T lymphocyte-associated protein 4 (CTLA-4), against HCC in 2008 (9), subsequent discoveries have unveiled a series of immune checkpoints, including programmed cell death protein 1 (PD-1)/programmed cell death ligand 1 (PD-L1), lymphocyte activation gene 3 (LAG-3), and T cell immunoglobulin and mucin-domain containing-3 (Tim-3). Researchers have been actively investigating whether inhibitors targeting these checkpoints hold practical clinical significance for HCC treatment. However, the progress of immunotherapy for HCC has been hindered by the absence of early diagnostic methods for HCC and the unique immune microenvironment in the liver, which is an immune-tolerant organ (10). Despite notable advancements in HCC immunotherapy, particularly the approval of nivolumab for HCC treatment in 2017, a lot remains to be done. Therefore, this review summarises and prospects the current research progress achieved through the application of clustered regularly interspaced short palindromic repeats (CRISPR), ribonucleic acid sequencing (RNA-seq), and other novel technologies in HCC immunotherapy, focusing on aspects such as the immune microenvironment, biomarkers, and therapeutic targets.

## 2 Overview of the immune microenvironment in the liver

**Figure 1:**
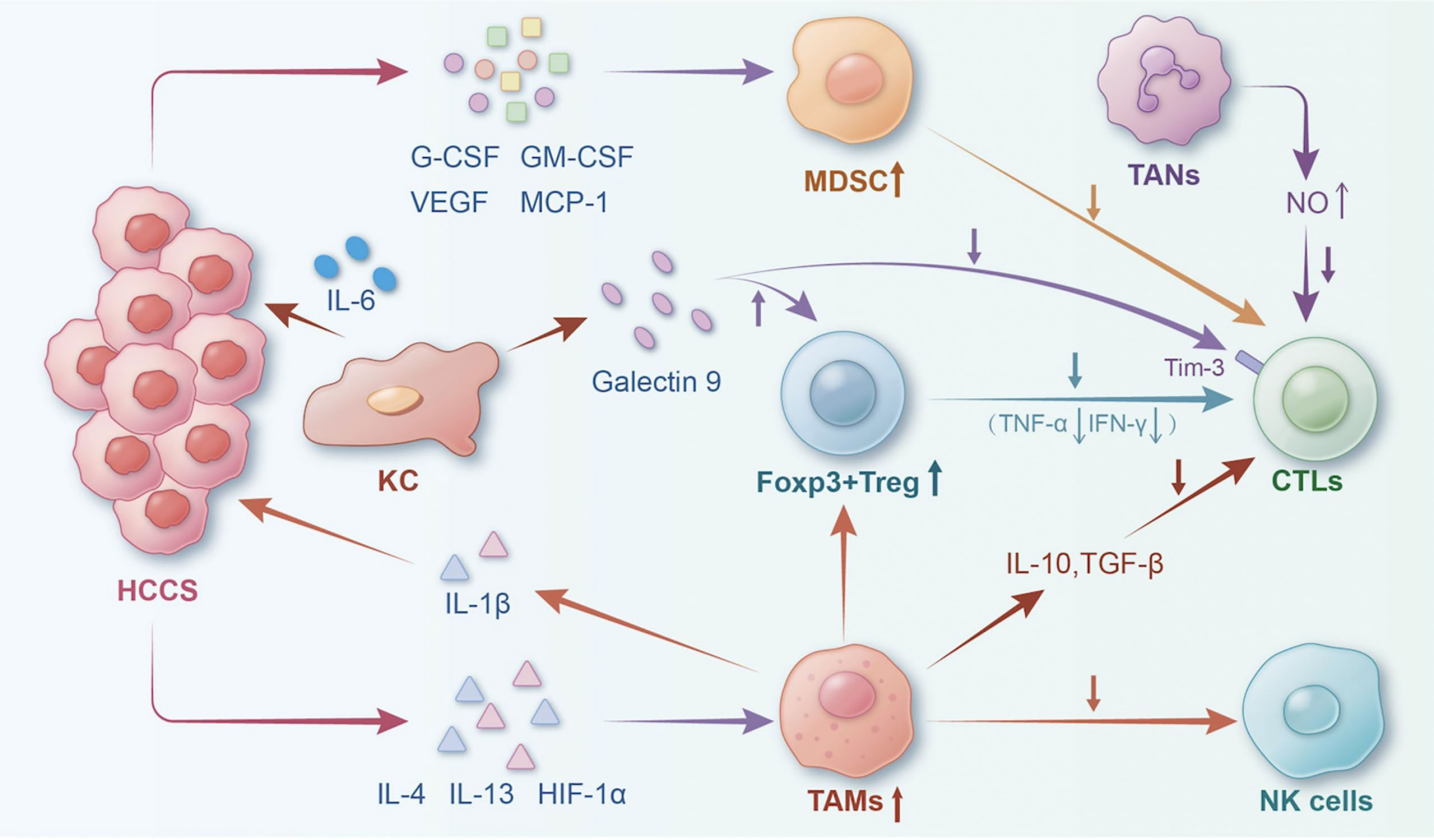
The landscape of the tumour immune microenvironment of HCC. The interaction of typical cells and factors in the HCC immune microenvironment could result in HCC progression. (Notes: HCCs, hepatocellular carcinoma; Tregs, regulatory T cells; TAMs, tumour-associated macrophages; TANs, tumour-associated neutrophils; CTLs, cytotoxic T lymphocytes; MDSCs, myeloid-derived suppressor cells; NK, natural killer cell; KC, Kupffer cell; HIF-1α, hypoxia inducible factor-1α; IL-1β, interlenkin-1β; IL-6, interlenkin-6).

The immune microenvironment of the liver exhibits distinct characteristics when compared with other organs. Being an immune-tolerant organ, the liver harbours several immunocompetent cells, including Kupffer cells (KCs), liver sinusoidal endothelial cells, and hepatic stellate cells as well as lymphocytes such as natural killer (NK), gamma-delta T cells, and dendritic cells (DCs) (11). The liver’s immune response is unique due to its chronic exposure to bacterial components and dietary antigens from the gastrointestinal tract. It must strike a balance between food tolerance and microbial antigens to prevent excessive inflammation caused by non-pathogenic antigens while maintaining the ability to respond rapidly and aggressively to pathogenic antigens and tumour cells (12, 13). This article primarily discusses the different cell types and cytokines present in the immune microenvironment, focusing on their role in inflammation and HCC development. (**Figure 1**).

### 2.1 KCs

For several years, KCs were regarded as potent weapons against liver cancer, exhibiting macrophage-like functions such as phagocytosis, antigen presentation, cytotoxicity, and cytokine secretion. On one hand, KCs can bind to HCC cells and present them to T cells, thereby enhancing the death of tumour cells (14). On the other hand, cytokines such as tumour necrosis factor-α (TNF-α) and interleukin (IL)-1β secreted by KCs can recruit cytotoxic T lymphocytes (CTLs) to target HCC cells. Zhang et al. (15) reported that KC-derived IL-10 could maintain immune homeostasis in the liver by inhibiting TNF-α and nitric oxide production by KCs. However, recent studies have focused on promoting the effects of KCs on HCC. It has been revealed that galectin-9, produced by KCs, is a natural ligand for Tim-3. Galectin-9 triggers the expansion of CD4^+^ CD25^+^ forkhead box P3 (FOXP3)^+^ CD127 (low) regulatory T cells (Tregs), the contraction of CD4^+^ effector T cells, and the apoptosis of CTLs in the HCC immune microenvironment, thereby promoting HCC development (16). Thus, further exploration of the mechanisms of action of KCs is crucial, considering their significance as a key cell in maintaining HCC immune microenvironment homeostasis.

### 2.2 Tumour-infiltrating lymphocytes

TILs play a crucial role in the antitumour immune response within solid tumours. They affect several cytokines and adaptive immune cells. Additionally, they modulate angiogenesis and innate immune response, thereby influencing HCC development. Several studies have demonstrated that a higher degree of lymphocyte infiltration in patients with liver cancer following surgery correlates with a lower recurrence rate and improved prognosis (17). Consequently, TILs have long been regarded as key components of the body’s immune response (17). However, Gao et al. (18) reported no correlation between CD3^+^, CD4^+^, CD8^+^, TILs, and OS or disease-free survival. Instead, the balance between Tregs and cytotoxic T cells within the tumour is a promising independent predictor of survival and HCC recurrence. Therefore, it suggests that the prognosis of cancer is the outcome of collective interactions among various cells in the tumour microenvironment.

### 2.3 CTLs

The CTLs in HCC are primarily CD8^+^ T cells. Within the tumour microenvironment, CD8^+^ T cells are stimulated by specific signals, which transform them into CTLs capable of producing interferon-γ (IFN-γ). This IFN-γ production aids in promoting inflammatory cytokine production and eliminates tumour cells. Notably, HCC tumours exhibit a significant infiltration of CD8^+^ CXC motif chemokine receptor 5 (CXCR5)^+^ T cells, and their presence is indicative of a favourable outcome (19). Moreover, T cells with CD8^+^ CXCR5^+^ receptors produce IL-21, which prompts B cells to differentiate into immunoglobulin (Ig) G-producing plasmablasts, thereby contributing to humoral immunity in HCC (19). However, a recent study has highlighted the role of immune checkpoint signalling in inducing an inhibitory effect on CTLs. The upregulation of inhibitory pathways, such as PD-1 and LAG-3, synergistically contributes to autoantigen and tumour antigen tolerance, thereby resulting in severe exhaustion of CTLs (20, 21).

### 2.4 Tumour-associated macrophages

TAMs are immune cells that inhibit antitumour immunity while promoting tumour progression and facilitating evasion from the tumour microenvironment (22). TAMs exist in two distinct phenotypes: M1-type TAMs, which are tumour suppressors and M2-type TAMs, which are tumour promoters. M1-type TAMs are induced by IFN-γ and/or lipopolysaccharides, enabling them to combat pathogens and control tumours. M2-type TAMs are induced by IL-4 and IL-13, leading to the release of immunosuppressive cytokines such as arginase 1, transforming growth factor-β, and IL-10, which promote tumour development while suppressing the immune response (23). M2-type TAMs produce IL-10, which is a potent inhibitory mediator. In HCC, besides IL-10 released by TAMs impairs the cytotoxicity of downstream CD8^+^ T cells and NK cells, increases the frequency of FOXP3+Tregs within tumours, and inhibits the activation of CD4^+^ CD25^−^ T cells (24, 25). The immunosuppressive effect of TAMs underscores the importance of investigating TAMs when studying tumourigenesis mechanisms and immunotherapy.

### 2.5 Tumour-associated neutrophils

The HCC infiltrated by TANs is associated with poor clinical outcomes, similar to TAMs. TANs can be classified into two types: N1 and N2. N1-type TANs possess cytotoxic properties that can inhibit tumour development, whereas N2-type TANs possess strong immunosuppressive abilities that promote tumour development (26). Exposure to TANs can induce a more malignant phenotype in HCC cells, enhancing their stemness, and attracting immunosuppressive macrophages and Tregs (27, 28). Furthermore, TANs contribute to the inhibition of antitumor immunity by producing nitric oxide through TNF-α activation (29). Overall, TANs play a significant role in immunosuppression within the tumour microenvironment of HCC. However, further investigation is required to elucidate their relationship with HCC components.

### 2.6 Tregs

Tregs, a subset of CD4^+^ T cells, play a crucial role in suppressing the immune response during HCC development. Tregs recruit and inhibit tumour-specific T cell activity within the tumour microenvironment. This promotes immune tolerance in tumour cells, allowing them to evade immune surveillance and clearance. In the context of HCC, Tregs could impair the function of CD8^+^ T cells by inhibiting the CD8^+^ T cell effector functions, including degranulation, perforin production, and granzyme production (30). The effect of Tregs on HCC treatment suggests that certain immunotherapeutic approaches might be less effective in HCC patients with significant Treg infiltration (31). Therefore, it is crucial to acknowledge the significant role of Tregs in HCC treatment.

### 2.7 Myeloid-derived suppressor cells

MDSCs constitute a heterogeneous population of immature myeloid cells that play a crucial role in promoting immunosuppression and angiogenesis. MDSCs can enhance the cancer stem cell gene expression, thereby promoting cancer stemness, metastatic potential, and tumourigenicity (32). MDSC infiltration is induced by various tumour-derived cytokines, such as granulocyte colony-stimulating factor, granulocyte-macrophage colony-stimulating factor, vascular endothelial growth factor, and monocyte chemotactic protein 1 (33). Liu et al. (34) observed that an increase in the number of monocytic MDSCs was associated with a decrease in TILs and an increase in tumourigenicity. In the context of HHC treatment, MDSCs exhibited significant reduction following combined radiotherapy and IL-12 therapy, consequently enhancing the manageability of HCC (35). Hence, further investigation of MDSCs is imperative to develop novel therapeutic strategies for liver cancer.

### 2.8 Main cytokines associated with HCC development

In the context of liver injury or infection, a robust protective response is initiated by a network of cytokines, chemokines, and growth factors involved in various interconnected inflammatory signaling pathways, the aberrant regulation of these pathways can result in the development of liver cancer. Simultaneously, the immune microenvironment of liver cancer facilitates the interaction among diverse immune cells *via* the intricate and interdependent secretion of cytokines.

In their investigation of the anoxic microenvironment of liver cancer, Zhang et al. (36) discovered that hypoxia inducible factor-1α (HIF-1α), a crucial transcriptional factor associated with hypoxia and inflammation, can stimulate the excessive expression of IL-1β in TAM. Furthermore, the study revealed that IL-1β can induce the production of HIF-1α in HCC. The overexpression of IL-1β has been found to facilitate the process of epithelial-mesenchymal transition (EMT) in HCC. Additionally, the release of IL-1β was observed to be augmented by the presence of necrotic HCC cell debris, which activated TAMs through the TLR4/TRIF/NF-κB signaling pathway. The positive feedback mechanism ultimately facilitated the onset of EMT and stimulated HCC cells to undergo metastatic progression.

The study conducted by Willscott E Naugler (37) and colleagues aimed to examine the sex-based disparities in HCC by administering diethylnitrosamine (DEN), a chemical carcinogen, to mice. The exposure to DEN was found to stimulate the production of IL-6 in KCs in a MyD88-dependent manner, thereby facilitating the onset of HCC. The incidence of this reaction was observed to be higher in male mice. The implementation of this pathway significantly mitigate the gender disparity in the incidence of HCC. The present study effectively elucidated the crucial involvement of IL-6 in the pathogenesis of HCC.

Yuan et al. (38). published a study indicating that lncRNA-activated by TGF-β (lncRNA-ATB) facilitated the upregulation of ZEB1 and ZEB2 through competitive binding to miR-200, ultimately leading to EMT and invasion. Furthermore, the lncRNA-ATB facilitated the organ colonization of disseminated tumor cells through binding IL-11 mRNA, autocrine induction of IL-11, and triggering STAT3 signaling. The significant contribution of these cytokines in the proliferation and dissemination of HCC warrants further investigation for potential clinical implementation.

## 3 HCC biomarkers

The implementation rate of early cancer detection programs for HCC remains low, and the recommended surveillance tools are not performing optimally (39). Furthermore, the gold standard of surveillance method (i.e., abdominal ultrasound with or without serum alpha-fetoprotein), fails to detect approximately 63% of early-stage tumours (39). Cholangiocarcinoma diagnosis encounters similar challenges. The use of biomarkers is a crucial aspect of managing patients with cancer as it can improve survival rates and optimise treatment approaches. There are three specific clinical areas, namely, risk stratification and early detection, prognosis prediction, and response prediction, where the demand for biomarkers is particularly urgent (40).

Following the Food and Drug Administration (FDA) approval of sorafenib (41), several systemic agents, mostly tyrosine kinase inhibitors, have been studied in phase 3 trials as first- and second-line treatments, resulting in improved survival rates (1). However, unlike sorafenib, PD-1 immune checkpoint inhibitors (ICIs), such as nivolumab (42) and pembrolizumab (43), did not meet their primary endpoints in phase III trials (44, 45). Consequently, extensive research efforts have been dedicated to identifying biomarkers that can predict the response to ICIs. Currently, multiple biomarkers have been identified that hold the potential in predicting a patient’s response to an ICI, which are further summarised below.

### 3.1 CD8^+^ PD-1^+^ CD161^+^ T cells

RNA-seq is a next-generation sequencing technique that allows for the analysis of RNA presence and quantity in a biological sample, providing insights into the dynamic changes in the cellular transcriptome (46, 47).

Li et al. (48) compared the differential expression levels of 35 surface markers and performed a diffusion map analysis. They found that CD8^+^ PD-1^+^ CD161^+^ T cells exhibited a proliferative and active phenotype, while CD8^+^ PD-1^+^ CD161^−^ T cells exhibited a progressively exhausted and aged phenotype. Deep single-cell RNA-seq (scRNA-seq) analysis revealed that the co-expression of PD-1, T cell immune receptor with Ig and immunoreceptor tyrosine-based inhibitory motif domains, and LAG-3 indicated the exhausted state of CD8^+^ PD-1^+^ CD161^−^ cells. Through multiplex immunofluorescence staining, they demonstrated that higher levels of CD8^+^ PD-1^+^ CD161^+^ T cells in non-tumour adjacent tissues were associated with a favourable outcome following tumour resection. Furthermore, patients with an abundance of CD8^+^ PD-1^+^ CD161^+^ T cells near the cancer site showed improved response to anti-PD-L1 treatment. These findings provide compelling evidence that this cell holds potential as a prognostic indicator.

### 3.2 PD-L1

**Figure 2:**
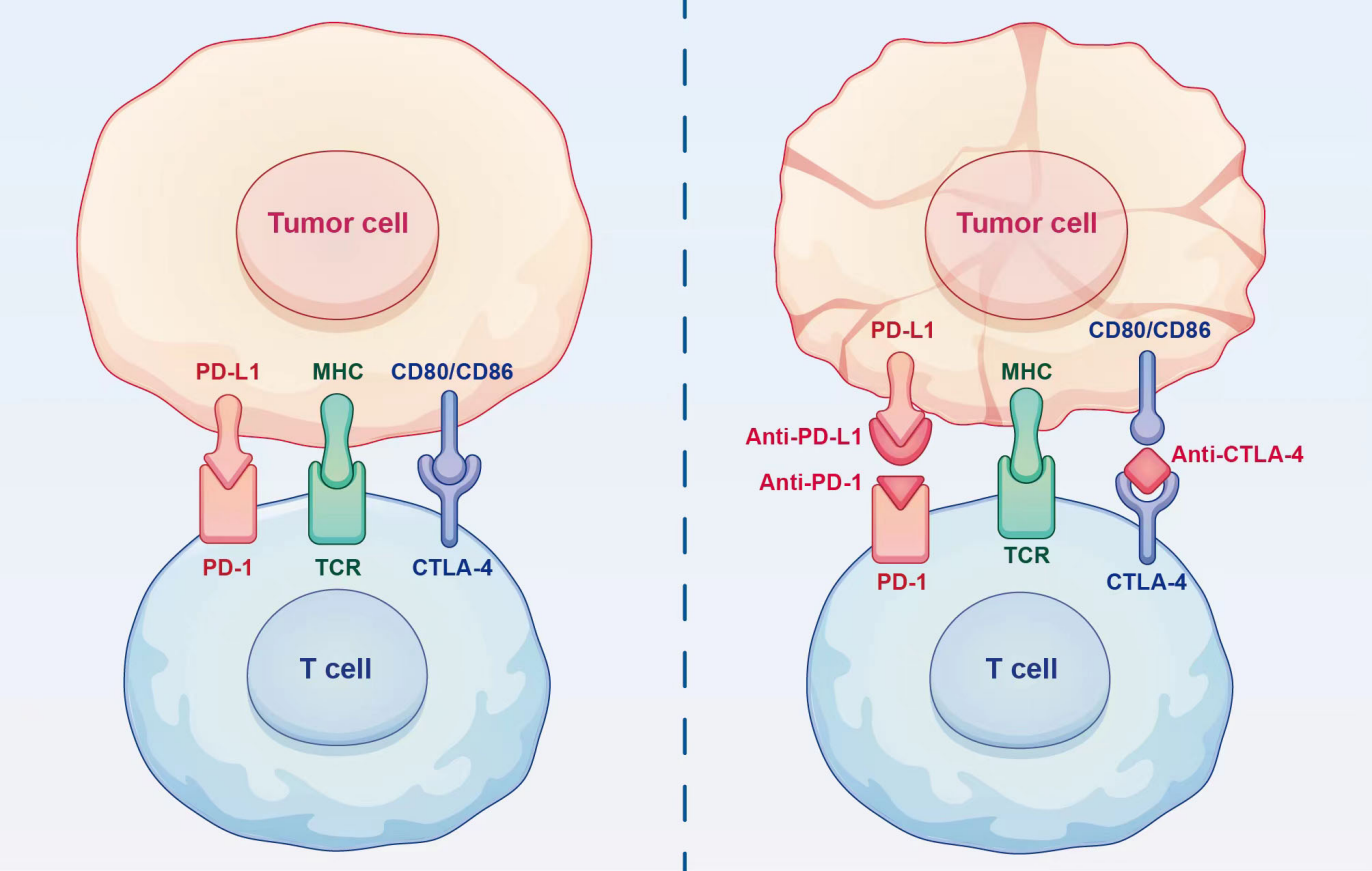
Mechanism of action of immune checkpoint inhibitors. Immune checkpoint inhibitors can restore the ability of T cells to eliminate tumour cells by blocking the binding of immune checkpoints to ligands.

Theoretically, the efficacy of PD-1/PD-L1 inhibitors could be predicted based on the PD-L1 expression (**Figure 2**). However, the complexity of the tumour microenvironment in HCC, including the presence of hepatitis and cirrhosis, as well as the classification of PD-L1, might undermine the reliability of PD-L1 as a predictor for the effectiveness of ICIs (49). The findings from CheckMate 040 (42) and CheckMate 459 (50) trials highlight the need for further investigation to determine the relationship between baseline PD-L1 expression levels and treatment benefits in HCC.

### 3.3 Tumour mutation burden

TMB refers to the total number of somatic gene coding errors, base substitutions, gene insertions, or deletions detected per million bases (51). Kim et al. (52) reported that a higher TMB was associated with an increased number of neoantigens that can be recognised by T cells, resulting in improved immunotherapy outcomes. Tumour-specific mutations produce highly immunogenic neoantigens, rendering tumour types with a high TMB more receptive to immunotherapy, thereby improving the treatment outcomes for patients. Therefore, neoantigens can serve as biomarkers and targets of tumour immunotherapy for predicting efficacy (53).

### 3.4 Circulating tumour deoxyribonucleic acid

In HCC or metastatic cancer cells, ctDNA carries comprehensive mutational information. It provides a precise and sensitive reflection of the tumour burden and enables the prediction of treatment response (54). It is known that patients with cancer have circulating free DNA in their plasma. In recent years, there has been significant interest in using ctDNA for specific clinical purposes, including early cancer diagnosis, prediction of treatment efficacy, and monitoring of metastasis.

The somatic mutations observed in HCC primarily stem from three key factors: telomere integrity (telomerase reverse transcriptase promoter, 55%), cell cycle regulation (tumour protein 53, 30%), and Wnt signalling (catenin beta 1, 30%) (55). Another study (56) reported that the frequency of these somatic mutations remains consistent in advanced HCC and early-stage HCC, suggesting their potential as predictive markers for primary drug resistance to systemic therapy. However, the number of patients with HCC enrolled in ctDNA clinical trials remains limited, and further investigation is required to establish the correlation between ctDNA and HCC progression.

## 4 Therapeutic targets for HCC

The investigation of the tumour microenvironment and immunotherapy in patients with HCC has been ongoing for several years. However, the research progress has not been satisfactory, and the clinical outcomes of immunotherapy in HCC have been suboptimal. Multiple factors might have contributed to these outcomes; however, one crucial aspect is the reduced immunogenicity of hepatocellular tumours. First, the liver exhibits immune tolerance due to exposure to dietary and gastrointestinal antigens (57). Second, the continuous stimulation of *de novo* antigens in HCC, along with the presence of immunosuppressive cell populations in the tumour tissues, creates an immunosuppressive tumour microenvironment. Moreover, patients with HCC tend to have an elevated number of Tregs in liver cancer tissues compared with normal tissues, and a higher Treg count is associated with a poorer prognosis (58, 59). Tregs can inhibit the function of CD8^+^ T cells, which monitor and eliminate tumour cells. Additionally, Han et al. (60) identified a new subset of regulatory CD14^+^ CTLA-4^+^ DCs in patients with HCC that produce high levels of IL-10 and indoleamine 2, 3-dioxygenase to inhibit the immune response of T cells. Furthermore, continuous antigen stimulation leads to the up-regulation of co-inhibitory signalling molecules such as CTLA-4, PD-1, and LAG-3 on tumour-specific lymphocytes, rendering T cells unresponsive (61, 62). Therefore, finding strategies to overcome the immunosuppressive microenvironment in HCC and using new technologies to identify therapeutic targets for HCC have become crucial research objectives that require attention.

### 4.1 Aryl hydrocarbon receptor

**Figure 3:**
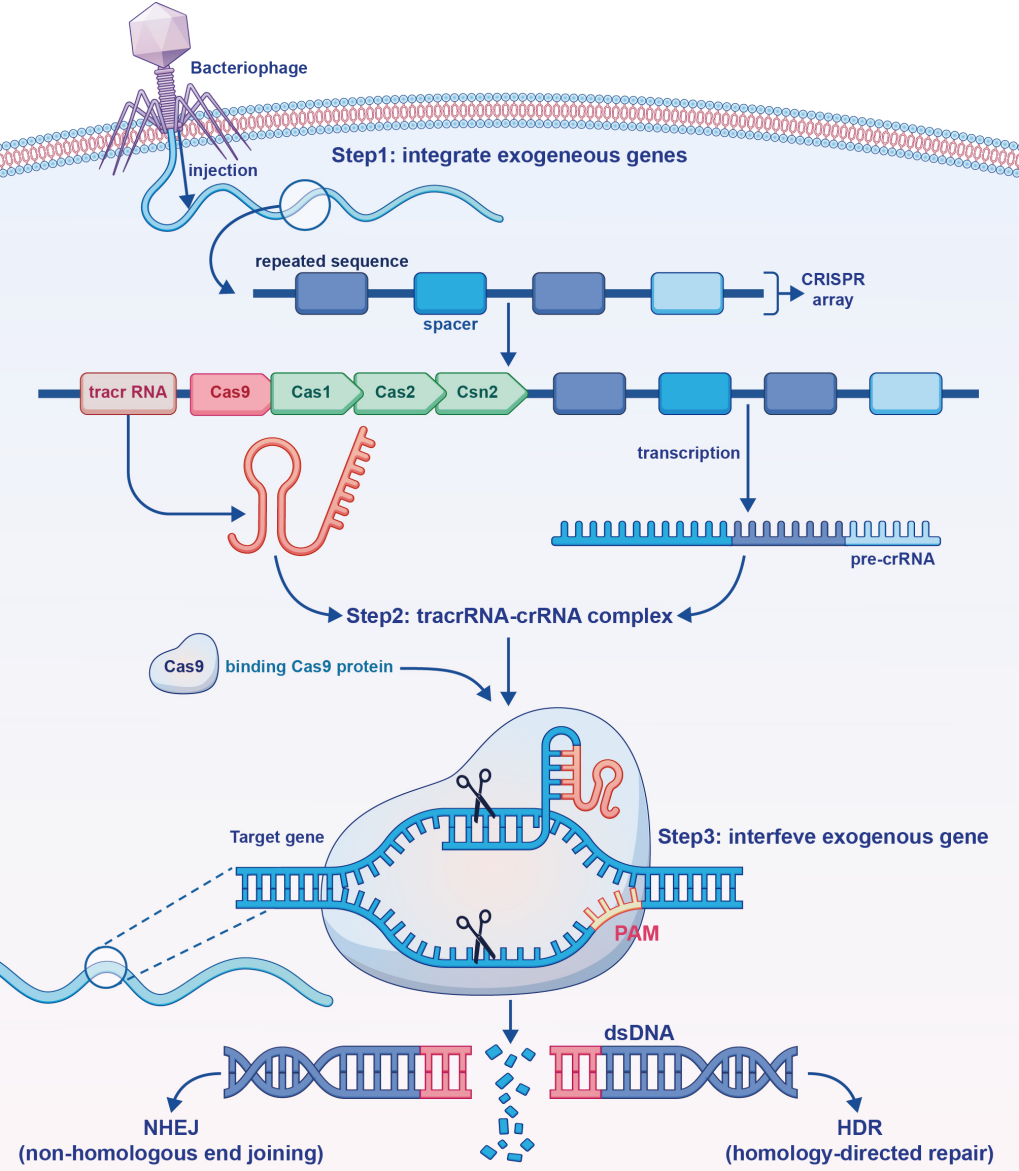
The mechanism of action of the clustered regularly interspaced short palindromic repeats (CRISPR)-CRISPR-associated protein 9 (Cas9) gene editing tool. Exogenous deoxyribonucleic acid (DNA) is processed and integrated into the CRISPR array. Trans-activating CRISPR ribonucleic acid (RNA) formed by transcription and pre-CRISPR RNA bind to each other, and form a complex with Cas9 formed by translation. Through base pairing, exogenous genes in the DNA sequence are specifically bound and excised.

DNA sequences called CRISPRs are found in the genomes of bacteria and archaea (63). CRISPR-associated protein 9 (Cas9) uses CRISPR sequences as a guide to cleave specific DNA strands that are complementary to these sequences. CRISPR-Cas9 can be used to edit genes within living organisms (**Figure 3**).

Primary HCC is one of the most common malignancies worldwide, and aflatoxin serves as a significant risk factor in its occurrence and progression. Using CRISPR-Cas9 genetic screens, Zhu et al. (64) identified targets for aflatoxin B1 (AFB1), with AHR being among the most notable findings. The formation of AFB1 adducts (cytochrome P450 metabolises AFB1 to AFB1-8, 9 epoxides, and subsequently reacts with DNA to form adducts) plays a crucial role in the development of aflatoxin-induced HCC. Immunofluorescence staining conducted by Zhu et al. demonstrated a significant reduction in the AFB1 adduct levels in AHR knockdown cells, indicating that AHR is targeted by AFB1. Additionally, the AHR knockdown cells exhibited enhanced tolerance to high concentrations of AFB1. Furthermore, patients with HCC expressing high levels of AHR exhibited improved responses to anti-PD-L1 therapy, suggesting that this treatment approach holds promise for AFB1-related HCC. Consequently, AHR can serve as a potential marker for PD-L1-based immunotherapy in patients with HCC.

### 4.2 CD74

CD74, a major histocompatibility complex (MHC) class II chaperone, was originally identified in MHC class II-positive cells, such as DCs, monocytes, macrophages, and B cells, among others (65). Recent studies have indicated that CD74 holds promise as a potential therapeutic target for malignant tumours (65, 66). In a study focusing on HCC, Xiao et al. (67) observed a significant distribution of CD74 in macrophages and found a positive correlation between CD74^+^ macrophage infiltration and CD8^+^ T cell activation *via* scRNA-seq and immunohistochemical analysis. Additionally, blocking CD74 resulted in impaired CD8^+^ CTL proliferation and antitumour activity. Therefore, CD74 emerges as a prognostic indicator for patients with HCC patients and might serve as a biomarker and potential therapeutic target.

### 4.3 PD1/PDL1

PD-L1 is a crucial ligand for PD-1. Within human HCC tissues, CD8^+^ T cells and KCs express high levels of PD-1 and PD-L1 (68). The interaction occurs when PD-1 on the surface of CD8^+^ T cells binds to PD-L1 on the surface of KCs. Subsequently, tyrosine residues within the cytoplasmic domain of PD-1 become phosphorylated, resulting in the recruitment of Src homology 2 domain-containing protein-tyrosine phosphatase-2 (SHP-2). SHP-2 then dephosphorylates key proteins, such as ζ chain-associated protein kinase 70 and phosphatidylinositol 3-kinase, downstream of the T cell receptor (TCR) and CD28 (69). This inhibits the cytotoxicity mediated by effector T cells and results in their functional impairment.

High PD-L1 expression in HCC is associated with a poorer prognosis (68). This observation suggests that targeting the PD-L1/PD-1 immune checkpoint could be a viable strategy for HCC treatment. In a study by Lu et al. (70), The CRISPR-Cas9 tool was used to cut the gene encoding PD-1 in non-small cell lung cancer immune cells. These modified immune cells were then expanded *in vitro* and reinjected into the patient, demonstrating an enhanced ability of the treated immune cells to respond to tumour cells. A similar approach can be used to study PD-1/PD-L1 target-based treatment for HCC.

Nivolumab, a monoclonal antibody targeting PD-1/PD-L1, functions by restoring the antitumour abilities of T cells (71). Data from the CheckMate 040 study, which evaluated nivolumab as a first-line therapy for advanced HCC demonstrated an objective response rate (ORR) of 20% and a mean OS of 28.6 months (42). In the CheckMate 459 study, a randomised controlled trial comparing nivolumab with sorafenib (44), it was found that although first-line treatment with nivolumab did not significantly improve OS compared with sorafenib, it exhibited a lower incidence of grade 3 to 4 treatment-related adverse events and improved quality of life in patients with advanced HCC. Consequently, nivolumab might serve as an alternative treatment option for patients with HCC who cannot undergo tyrosine kinase inhibitor therapy or antiangiogenic therapy.

### 4.4 CTLA-4

CTLA-4, a CD28 homolog, inhibits T cell response by directly delivering an inhibitory signal to the T cell (72). The CTLA-4 protein alters intracellular T cell signalling and prevents CD28 from binding to CD80 and CD86, which is essential for optimal T cell activation (73, 74). Furthermore, studies have revealed that the presence of CTLA-4 decreases helper T cell activity while increasing Treg activity (75). Tregs rely on CTLA-4 to suppress immune responses by inhibiting the ability of antigen-presenting cells to activate other T cells. Additionally, depleting CTLA-4 in Tregs affects their suppressor function and promotes tumour immunity (76, 77). Despite these findings, the anti-CTLA-4 monoclonal antibody tremelimumab did not demonstrate significant clinical effects (9). Therefore, further investigation is warranted to explore immunotherapy regimens targeting CTLA-4.

### 4.5 Other new targets

LAG-3 serves as an important inhibitory immune checkpoint. CD8^+^ T cells express LAG-3, which binds to MHC class II molecules (78). Studies have demonstrated that the up-regulation of LAG-3 expression is associated with T cell effector dysfunction (79). Additionally, blocking LAG-3 expression has been shown to enhance the proliferation of CD8^+^ and CD4^+^ TILs *in vitro* and affect cytokine production (80). Regarding LAG-3 target-based therapy, Zhou et al. (80) reported that T cells respond to HCC tumour antigens when treated with antibodies against PD-L1, Tim-3, or LAG-3. Combining these antibodies might offer additive effects, suggesting the potential for advancing HCC treatment research.

TAMs express Tim-3, which promotes HCC growth (81). Large numbers of Tim-3^+^ TILs and Tim-3^+^ TAMs in HCC lesions are associated with reduced survival and increased recurrence, leading to a poor prognosis (82). Similar to LAG-3, the blockade of Tim-3 targets can enhance the function of effector T cells, promote cytokine release, and inhibit tumour progression (20). However, the exact efficacy of targeting Tim-3 in HCC treatment remains uncertain. Ongoing research is investigating the use of the anti-Tim-3 antibody Cobolimab (83).

## 5 Treatment strategies for HCC

The primary treatment methods for HCC include surgical resection and local ablation (84); however, they have certain limitations. Surgical resection is often associated with a high recurrence rate, ranging from 40% to 70% after surgery (1, 85). Additionally, most HCC cases are diagnosed at an advanced stage, often accompanied by cirrhosis and other diseases, making surgical treatment unsuitable for these patients. Therefore, the identification of new and effective treatment strategies is crucial. Immunotherapy has emerged as a breakthrough in the treatment of advanced liver cancer since 2017 (86). It aims to activate the immune system, modify the tumour immune microenvironment, and use the body’s immune function to treat tumours. Previous studies have focused on monotherapy targeting key molecules in HCC, such as PD-1, CTLA-4, and LAG-3. This section primarily highlights the advancements and efficacy of ICIs in combination therapy for HCC.

### 5.1 Combination therapy

Compared to monotherapy with ICIs, combination therapy can target multiple immune checkpoint pathways, resulting in improved patient outcomes. However, it is essential to consider not only the increased efficacy associated with combination therapy but also its clinical significance and potential for increased drug toxicity and immune-related adverse events. The Checkmate 040 trial (87) evaluated the safety and efficacy of nivolumab plus ipilimumab in patients with advanced HCC treated with sorafenib. The overall ORR and disease control rate were 31.0% and 49.0%, respectively, and the combination regimen demonstrated acceptable safety. Consequently, the FDA approved this regimen in 2020 for patients with advanced HCC who had previously received sorafenib. In a phase I/II trial (NCT02519348), tremelimumab plus durvalumab (T300+D) was compared with tremelimumab or durvalumab monotherapy (88). The results revealed a median OS (95% confidence interval [CI]) of 18.7 (10.8–27.3) months and a confirmed ORR (95% CI) of 24.0% (14.9–35.3) for the T300+D regimen, indicating superior efficacy compared to monotherapy. Moreover, the T300+D regimen exhibited higher levels of CD8+ lymphocyte proliferation while maintaining acceptable tolerability compared with monotherapy. These studies suggest that combination regimens with ICIs demonstrate a promising benefit-risk profile and hold the potential for advancing therapeutic strategies for HCC.

Furthermore, ICIs can be combined with other treatment modalities, including targeted therapy. In a phase III study of IMbrave150 conducted in May 2020, the combination of atezolizumab and bevacizumab (A+T) was superior to sorafenib monotherapy (89). Similarly, the IMbrave150 China cohort results, published during the European Association for the Study of the Liver summit in the same year suggested that the A+T regimen might be particularly suitable for the Chinese population (90). ICIs can also be used in conjunction with transarterial chemoembolisation (TACE). In a phase I trial combining ICIs and TACE, the efficacy of nivolumab plus drug-eluting bead TACE was demonstrated, showing a partial response of 22%, a stable disease rate of 78%, and a 12-month OS rate of 71% in nine patients.

TGF-β serves a dual role in solid tumor progression by promoting EMT and creating a conducive tumor microenvironment for growth and metastasis (91). Recent preclinical investigations have demonstrated that TGF-β impedes the efficacy of tumor immunotherapy by hindering T cell infiltration into the tumor center. However, the combination of TGF-β blockade and anti PD-L1 antibodies exhibits a clear antitumor synergy (92). In a phase II trial, Galunisertib (LY2157299), a TGF-βR1 inhibitor, exhibited satisfactory safety and extended overall survival outcomes when administered in conjunction with sorafenib, indicating its efficacy as a second-line treatment for hepatocellular carcinoma (93). Presently, a clinical trial is underway to evaluate the effectiveness of combining Galunisertib with nivolumab (anti-PD-1) for the treatment of relapsed or refractory HCC with elevated alpha-fetoprotein (AFP) levels of ≥200 ng/mL (NCT02423343). In preclinical trials, M7824, a bifunctional checkpoint inhibitor targeting both PD-L1 and the extracellular domain of TGFβR2 (TGF-β trap), promotes the activation of CD8 T cells and NK cells and has demonstrated superior results compared to monotherapy with anti-TGF-β or anti-PD-L1 in preclinical trials (94). Preliminary data from two dose-escalation phase I studies (NCT02699515, NCT02517398) indicate that M7824 demonstrates a manageable safety in patients with advanced solid tumors, including hepatocellular carcinoma (95, 96). Another PD-L1/TGF-β bispecific antibody, Y101D, is currently being evaluated in a phase 1 clinical trial targeting solid tumors (NCT05028556).

Immune checkpoint inhibitors in combination with chemotherapy can synergistically enhance the efficacy of various malignant tumors. In an open-label, multicenter phase Ib/II study (NCT03092895), treatment-naïve patients with advanced primary hepatocellular carcinoma received camrelizumab (anti PD-1) in combination with FOLFOX4 (fluorouracil+calcium folinate+oxaliplatin) or GEMOX (gemcitabine and oxaliplatin) regimen showed good safety, tolerability and preliminary antitumor activity (97). Meanwhile, a prospective, randomized, double-blind, multicenter phase III study to determine the efficacy and safety of camrelizumab plus FOLFOX4 versus placebo plus FOLFOX4 in HCC (NCT03605706) is currently ongoing.

Additionally, ICIs can be combined with radiotherapy and other treatment modalities, which are beyond the scope of this discussion.

### 5.2 Hotspot therapy — chimeric antigen receptor T cells

In cancer immunotherapy, CRISPR-Cas9 technology has revolutionised the generation of CAR-T, which represents a remarkable application of this technology. CAR-T immunotherapy involves the genetic modification of T cells using lentiviruses or retroviruses. These genetically modified T cells express CARs and undergo significant expansion upon recognition of specific antigens *in vitro* (98, 99). After reaching the desired number of treatments, these T cells are adoptively reinjected into the patient. CAR-T cells with CARs, can penetrate the complex tumour microenvironment and target high-affinity antigens on the tumour cell surface to kill the tumour cell. However, inhibitory surface receptors on T cells, such as PD-1, CTLA-4, and LAG-3, can impede the efficacy of CAR-T. CRISPR-Cas9 can knock down the genes encoding these surface receptors and inhibit their effects, thereby enhancing the effector capacity of CAR-T. Guo et al. (100) demonstrated that disrupting endogenous PD-1 expression using CRISPR-Cas9 technology improved the antitumour activity, invasiveness, and persistence of CAR-T, thereby yielding significant therapeutic benefits in HCC treatment. Additionally, Zhang et al. (101) successfully constructed CAR-T with LAG-3 receptor knockout, suggesting that exploring the construction of CAR-T with a double knockout of PD-1 and LAG-3 might result in improved outcomes. Considering that xenoinhibitory rejection can be triggered by human leukocyte antigen-I (HLA-I) on the surface of T cells, Ren et al. (102) developed universal CAR-T by genetically modifying them to have defective TCR and HLA-I expression, effectively preventing xenograft rejection. This universal CAR-T can be used for multi-patient therapy, exhibiting potent antitumour properties.

## 6 Future perspectives

This article provides an overview of the current research trends and advancements in identifying novel biomarkers and therapeutic targets for HCC. Additionally, the immunotherapy strategies commonly used for HCC, such as CAR-T therapy, were briefly reviewed. Based on the existing ICIs, this study aimed to explore more effective drug utilisation and clinical treatment strategies. In a phase Ib trial involving 100 patients with untreated, unresectable HCC, a combination of the multikinase inhibitor lenvatinib and the ICI pembrolizumab demonstrated excellent antitumour efficacy with an ORR of 46%, a median PFS of 9.3 months, and a median OS of 22.0 months (103). The enrolment for a phase III trial comparing this regimen to lenvatinib monotherapy has been completed (LEAP-002; NCT03713593), which will further evaluate the safety, therapeutic efficacy, and risk-benefit profile of this protocol. Additionally, early results from clinical studies, such as CheckMate 459 (44), comparing nivolumab vs. sorafenib (ORR 15% vs. 7%, median PFS 3.7 vs. 3.8 months) and IMbrave 150 (104), comparing atezolizumab plus bevacizumab vs. sorafenib monotherapy (ORR 30% vs. 11%, median PFS 6.8 vs. 4.3 months), have yielded promising outcomes. It is anticipated that these therapeutic strategies will pave the way for significant advancements in HCC treatment.

By employing comprehensive CRISPR-Cas9 screening throughout the entire genome, progress can be made in identifying novel and more effective immune checkpoints and immune targets that influence HCC progression. This approach allows us to explore the efficacy of clinical drugs targeting these checkpoints or targets with improved effectiveness. It is anticipated that untapped technologies, not yet applied to HCC research, could play vital roles in advancing HCC immunotherapy. One such technology is digital spatial profiling (DSP), which offers a superior research method compared with scRNA-seq for detecting gene expression levels and acquiring their spatial location *in situ* (105). This circumvents the loss of spatial heterogeneity in tumours (106). Notably, Brady et al. (107) used DSP to analyse the tissue cells from metastatic prostate cancer (mPC) and discovered that low CTLA-4 and PD-1/PD-L1 expression was consistent with the low reactivity of patients with mPC to ICIs. Furthermore, the high expression of CD276/B7-H3 and Tim-3 suggested that DSP holds promise for discovering potential immune targets. In the future, it is envisioned that DSP will be used to analyse immune cells *in situ* within the complex immune microenvironment of HCC, thereby uncovering novel immune checkpoints that are more conducive to HCC treatment.

## 7 Conclusions

HCC is a highly prevalent malignant tumour in China. Its unique immune microenvironment, coupled with the lack of early diagnostic methods, has posed challenges in developing effective clinical treatments for many years. Immunotherapy has emerged as a promising approach for advanced HCC and has garnered significant attention from researchers. In addition to known immune checkpoints (PD-1/PD-L1, CTLA-4, and LAG-3) and biomarkers (TMB and ctDNA), novel biomarkers and immune targets such as AHR, CD74, CD8^+^ PD-1^+^ CD161^+^ T cells have been discovered *via* CRISPR and scRNA-seq. These discoveries provide a new research direction for early diagnosis and treatment of HCC. Combinations of ICIs or ICIs with targeted therapy have demonstrated practical clinical efficacy in HCC treatment. Moreover, research efforts have focused on the CRISPR-based knockdown of genes encoding immune checkpoints to enhance the effectiveness of CAR-T, representing a significant research direction. In the future, CRISPR, DSP, and other emerging technologies might provide novel therapeutic approaches for HCC, instilling hope for improved survival outcomes among patients with HCC.

## Author contributions

Drafting the manuscript: DJ, XZ. Provision of study materials: BC, RW, XM. Conceptualization and Supervision: YL, XYZ. All authors contributed to the article and approved the submitted version.
